# Geographically associated endophytic fungi contribute to the tropane alkaloids accumulation of *Anisodus tanguticus*

**DOI:** 10.3389/fpls.2023.1297546

**Published:** 2023-11-30

**Authors:** Bo Wang, Chen Chen, Yuanming Xiao, Yan He, Ying Gao, Zongxiu Kang, Xiaoxuan Wei, Yujie Deng, Shihong Feng, Guoying Zhou

**Affiliations:** ^1^ CAS Key Laboratory of Tibetan Medicine Research, Northwest Institute of Plateau Biology, Xining, China; ^2^ University of Chinese Academy of Sciences, Beijing, China; ^3^ Datong Beichuan Heyuan District National Nature Reserve, Xining, China; ^4^ Chengdu Tianxianzi agricultural science and technology development Co., LTD, Chengdu, China

**Keywords:** Tibetan medicines, Anisodus tanguticus, tropane alkaloid, endophytes, plant-microbe interaction

## Abstract

*Anisodus tanguticus* is a valuable plant for extracting tropane alkaloids. However, the mechanisms by which plant microbiome mediate the accumulation of tropane alkaloids in *Anisodus tanguticus* are still not well understood. In this study, we collected 55 wild *Anisodus tanguticus* populations on the Tibetan Plateau and the tropane alkaloids content, and root-related bacteria and fungi diversity were analyzed using HPLC and 16 s rDNA and ITS sequencing. The results showed that tropane alkaloids content has obvious geographical distribution characteristics. Anisodine content had a significant positive correlation with latitude, while anisodamine and atropine content had a significant negative correlation with latitude. Variation partition analysis (VPA) showed that root endophytes play a significant role in promoting tropane alkaloid production in *Anisodus tanguticus* roots. The root endophytes alone explained 14% of the variation, which was the largest contributor. Soil properties variables could independently explain 5% of the variation, and climate variables could explain 1% of the variation. Of these, endophytic fungi alone accounted for 11%, while bacteria explained only 5%. Random forests and Mantel test showed that different regionally enriched endophytic fungi have a greater impact on the accumulation of tropane alkaloids than the whole endophytic fungi. Richness and relative abundance of enriched endophytic fungi in Hengduan-Qilian Mountains (HQ) group has a significant positive correlation with anisodine content, while richness and relative abundance of enriched endophytic fungi in Himalayas-Hengduan Mountains (HH) group has a significant positive correlation with anisodamine and atropine content. And, these enriched endophytic fungi have high network connectivity and distributed in separate network modules. This study further confirmed that endophytes were closely related to tropane alkaloids accumulation in *Anisodus tanguticus* and contribute to promote sustainable development, cultivation, and precision medicine of *Anisodus tanguticus.*

## Introduction

Tropane alkaloids are a group of alkaloids that have a pyrrole ring and a piperidine ring that form the basic skeleton of tropane (Tian et al., 2022). Tropane alkaloids exhibit anticholinergic properties by blocking the action of the neurotransmitter acetylcholine in both the central and peripheral nervous systems through binding to muscarinic and/or nicotinic receptors (Lockery et al., 2021). The distribution of tropane alkaloids in plants such as Convolvulaceae, Sequoiaceae, Solanaceae, etc. has been reported (Griffin and Lin, 2000). Among them, the highest concentrations of tropane alkaloids are present in Solanaceae (Shim et al., 2022). Tropane alkaloids from solanaceae plants, including anisodine, anisodamine, scopolamine, and atropine are used to treat muscle spasms, improve visual function, treat gastrointestinal and renal colic, anesthesia, shock, and poisoning by organophosphorus compounds (Fan et al., 2016; Xu and Zhao, 2017; Liu et al., 2019a; Yu et al., 2021). As a result, the market for drugs containing tropane alkaloids is growing rapidly. Antispasmodic drugs in China to be worth more than 3 billion yuan by 2020 (Zhou et al., 2023). Currently, there are no commercially viable methods of chemically synthesizing tropane alkaloids, which are mainly extracted from the medicinal plants (Hu et al., 2019; Kohnen-Johannsen and Kayser, 2019).

*Anisodus tanguticus*, a solanaceae herbaceous plant, is a tropane alkaloids-producing plant that was used as an anesthetic in in Tibetan medicine (Chen et al., 2022a). Its roots are rich in anisodine, anisodamine, and atropine. Many pharmaceutical companies extract anisodine, anisodamine, and atropine from *A. tanguticus* roots (For example, Chengdu First Pharmaceutical Co). Furthermore, a growing body of modern pharmacological research has shown that *A. tanguticus* also has anti-inflammatory, anti-oxidation, and anti-cancer activity (Yu et al., 2021). Due to *A. tanguticus* various medicinal value, a growing number of drugs consisted of *A. tanguticus* or its extracts, and the market demand for *A. tanguticus* is increasing.

It is well known that there are quality differences of medicines from different distribution origins (Liu et al., 2019b). Previous studies have predominantly examined the impact of external factors, including soil conditions and climate factors, on the formation of herb quality (Liu et al., 2021; Li et al., 2022; Liu et al., 2022). However, the influence of the internal environment of medicinal plants on the formation of herbal quality is receiving increasing attention. Endophytes, as the essential part of the inner environment of herbs, are a class of microorganisms that live all or part of their life history in cells of the tissues and organs or intracellular spaces of the healthy plant without any harmful effects on the host plants (Schulz and Boyle, 2005). During the long-term process of coevolution, endophytes have acquired the ability to produce the same or similar compounds as their host plants (Xu et al., 2009). For example, endophytic fungi isolated from Taxus plants can produce paclitaxel; endophytic fungi isolated from *Camptotheca acuminata* can produce camptothecin; endophytic fungi isolated from *Catharanthus roseus* can produce vinblastine and vincristine (Stierle et al., 1993; Ran et al., 2017; Das et al., 2020). Secondly, endophytes can affect plant metabolic pathways though elicitor effect or lateral gene transfer to promote the production of active ingredients in medicinal plants (Zhai et al., 2017). For example, Pseudocardiae YIM 63111 has been found to stimulate the production of artemisinin by regulating the genes associated with the biosynthesis of artemisinin (Li et al., 2012). However, large-scale investigative experiments have shown that the capacity of isolated endophytes from different origins, but belonging to the same herb, to produce or enhance the accumulation of secondary metabolites varies. The production capacity of cinchona alkaloids by endophytes isolated from Cinchona plants in Japan is lower compared to those from Indonesia (Maehara et al., 2019). This helps us to understand the mechanism of medicinal plants from different production areas quality formation from a new perspective.

Chen et al.’s research on *A. tanguticus* from Gansu, Sichuan, and Qinghai results suggested that there were significant differences in anisodamine, anisodine and atropine content of *A. tanguticus* from geographic areas (Chen et al., 2022a). However, A. tanguticus microbiomes and their relationship with secondary metabolites have not been reported. In this study, we investigated the tropane alkaloids and endophytes of 55 wild *A. tanguticus* in the Qinghai–Tibetan Plateau. The aim of this research is to (i) clarify the geographical distribution characteristics of tropane alkaloids of wild *A. tanguticus* populations in Qinghai–Tibetan Plateau; (ii) clarify the diversity and composition of root-related microbiome of *A. tanguticus*; (iii) explore the correlation between different tropane alkaloids and root-related microbiome of *A. tanguticus*.

## Materials and methods

### Sample collection

**Figure 1:**
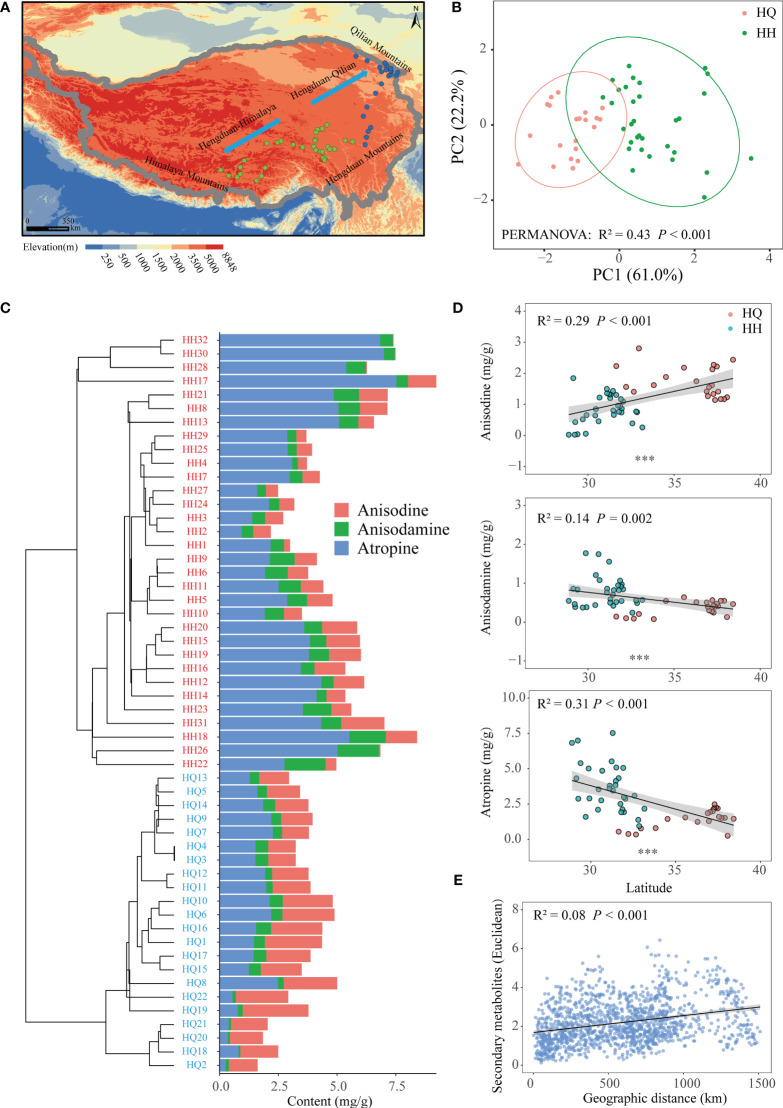
The geographic distribution pattern of tropane alkaloids of *Anisodus tanguticus*. **(A)** Map showing sampling sites in the Qinghai-Tibetan Plateau. **(B)** Principal-component analysis plot showing the distinct tropane alkaloids among samples of HH and HQ. **(C)** Hierarchical cluster analysis of tropane alkaloids of *Anisodus tanguticus*. **(D)** Regression analysis of tropane alkaloids content and latitude. **(E)** Regression analysis of tropane alkaloids content dissimilarity and geographical distance.

We collected 55 wild *A. tanguticus* populations on the Tibetan Plateau between August and September 2020 (**Figure 1A**; **Table S1**) (Wang et al., 2023). For each site, we randomly selected five *A. tanguticus* plants. Tightly adhering soil was collected as rhizosphere soil, and fine roots were collected. At each sampling locus, the five root and soil samples were mixed into one composite sample. The samples were all stored in a car refrigerator and then transported to the laboratory. The roots were sterilized with 70% alcohol and 0.5% sodium hypochlorite (Luo et al., 2021). All samples were stored at − 80°C for preservation.

### Soil physicochemical analyses

Total nitrogen (TN) and total carbon (TC) were analyzed using a Vario EL Elemental Analyzer (Elementar Analysensysteme GmbH, Germany). The total phosphorus (TP) was wet digestion with H_2_SO_4_ + HClO_4_, and then determined by the molybdenum antimony anti-colorimetric method (Bao, 2018). The soil available phosphorus (AP) was extracted with 0.5 M sodium bicarbonate, and then determined by the molybdenum antimony anti-colorimetric method (Bao, 2018). Soil ammonium nitrogen (NO_3_^–^N) and nitrate nitrogen (NH_4_^+^-N) were extracted with 2 M potassium chloride and then measured with the determined by an AQ1 discrete analyzer (Yu et al., 2022). Soil organic matter was determined by oxidization with 0.4 M of potassium dichromate–concentrated sulfuric acid solution.

### Tropane alkaloid extraction and determination

In brief, tropane alkaloids in *A. tanguticus* root were extracted from 0.2 g powder samples with 2% formic acid. The suspension was centrifuged for 10 minutes and then filtered through a 0.22 μm filter membrane for high-performance liquid chromatography (HPLC) analysis. Anisodine, anisodamine, and atropine were precisely measured and subsequently diluted with methanol to prepare stock solutions with concentrations of 0.36, 0.54, and 0.62 for anisodine, anisodamine, and atropine, respectively. All the commercial standards were purchased from Sigma–Aldrich (Madrid, Spain). Agilent 1260 instrument equipped with a VWD detector was used to determine the tropane alkaloids content (Agilent, USA). Samples were separated on a ZORBAX Eclipse Plus column (250 mm×4.6mm). The HPLC mobile phases were H_2_O with 0.1% trifluoroacetic acid (solvent A) and acetonitrile with 0.1% trifluoroacetic acid (solvent B). The elution gradient was 10% B to 10% B in 25 min; The detection wavelength was 215 nm. The assays were conducted in triplicate for accuracy and precision.

### Microbial DNA processing

We used a FastDNA SPIN Kit to extract Microbial DNA from the root and soil samples. Two primer sets, 799F (5′-AACMGGATTAGATACCCKG-3′)/1392R (5′-ACGGGCGGTGTGTRC-3′) and 799F (5′-AACMGGATTAGATACCCKG-3′)/1193R (5′-ACGTCATCCCCACCTTCC-3′), were utilized to amplify the V5-V7 region of the bacterial 16S rRNA gene (Zhang et al., 2022b). The ITS2 region of the fungal gene was amplified using the ITS3F (GCATCGATGAAGAACGCAGC) and ITS4R (TCCTCCGCTTATTGATATGC) primers (Liu et al., 2018). The PCR amplification was conducted in a 20-μL reaction mixture consisting of 10 ng of template DNA, 0.4 μL of FastPfu Polymerase, 4 μL of 5 × FastPfu Buffer, 2 μL of dNTPs (2.5 mM), and 0.8 μL of each primer (5 μM). The targeted region was amplified using the 799F and 1392R primers after an initial denaturation step at 95°C for 3 min. This was followed by 27 cycles of 95°C for 30 s, 55°C for 30 s, and 72°C for 45 s, with a final elongation step of 10 min at 72°C. For the second step, the 799F-1193R primers were used and the same conditions as the first step of the PCR were applied for 13 cycles. Fungal PCR reactions were carried out under identical conditions as the bacterial PCR reactions for 35 cycles. All samples were amplified in triplicate. The PCR product was extracted from a 2% agarose gel and purified using the AxyPrep DNA Gel Extraction Kit (Axygen Biosciences, Union City, CA, USA). DNA samples were sequenced on an Illumina MiSeq PE300 platform (Illumina, San Diego, USA) at Shanghai Majorbio Bio-pharm Technology Co., Ltd. QIIME software and the UPARSE pipeline were used to treat raw sequences. Operational taxonomic units (OTUs) were clustered at a 97% similarity level using UPARSE version 7.1. Taxonomic classification of OTUs was conducted using the SILVA database for the 16S rRNA gene and UNITE database 8.0 for the ITS region. All raw sequencing data have been submitted to the NCBI Sequence Read Archive (SRA) database under the accession numbers PRJNA837402 (16S) and PRJNA837692 (ITS).

### Statistical analysis

Statistical analysis was performed in the R program and visualized using the “ggplot2” package (Wickham, 2016; R Core Team, 2021). Based on the concentration of three tropane alkaloids, samples were grouped by hierarchical clustering using the function hclust in R (method = median). Principal component analysis (PCA) was also performed to assess the similarity of secondary metabolites between different groups. Statistical differences in the secondary metabolites between different groups were determined by PERMANOVA using the “vegan” package with 9999 permutations (Oksanen et al., 2011). Geographic distance between the site was calculated with the “geosphere” package. Secondary metabolite distance was calculated with the “vegan” package (Euclidean distance). The differences in OTU richness between different groups were assessed using Wilcox test. Non-metric multidimensional scaling (NMDS) was used to assess the similarity of the communities between different groups using the “vegan” package. We tested for differential OTU abundance between different groups using likelihood ratio tests (LRT) with the “edgeR” package (Robinson et al., 2010). |logFC| ≥ 2 and pvalue < 0.05 was the cut-off value.

We obtained the mean annual temperature (MAT) and mean annual precipitation (MAP) data at 30-arc seconds from the WorldClim (https://worldclim.org/) as climate variable. Geographic variables include geographic distance and altitude. Soil variables include total nitrogen, total phosphorus, available phosphorus, soil organic matter, ammonium nitrogen, and nitrate nitrogen. Because of a significant distinction in root endophytic bacterial and fungal communities between different groups, microbial variables selected endophytic bacterial and fungal alpha diversity and beta diversity. We included alpha diversity and beta diversity of enriched microbial OTUs for different groups as separate variables in the analysis. The 22 important environmental factors were used for random forest analysis to estimate the contribution of each variable to secondary metabolites variation with the “rfPermute” package (Archer, 2022). Variance partition analysis (VPA) was performed to estimate the contribution of soil conditions, climate factors, and endophytes to secondary metabolites variation using the “vegan” package.

We constructed co-occurrence networks to evaluate species coexistence across different regions. In order to rule out the influence of rare OTUs, the OTUs with more than 0.01% relative abundance were selected to calculate Spearman’s rank correlation coefficients. Spearman correlation coefficient r > |0.4| and *P* < 0.05 (FDR) were used for co-occurrence network construction with the ‘Hmisc’ package (Frank and Harrell, 2022). The networks were visualized using Gephi platform(https://gephi.org/). In order to describe the complexity of the network, the properties (the average path length, network diameter, average degree, and average clustering coefficient) were calculated. The difference in topological features between enriched microbial OTUs and others was assessed using Wilcox test.

## Results

### Geographical characteristics of tropane alkaloids of *A tanguticus*

In our study, we collected *A. tanguticus* roots and determined the anisodine, anisodamine and atropine content. The content of anisodine was 1.19 mg/g; the content of anisodamine was 0.61 mg/g and the content of atropine was 2.76 mg/g (**Table S2**). Based on the concentration of three tropane alkaloids, hierarchical clustering indicated that these samples were divided into two groups (**Figure 1C**). Hengduan Mountains south to the Himalayas for one group (HH), and north to the Qilian Mountains for another group (HQ) (**Figure 1A**). PCA also showed a clear distinction between HQ and HH groups (Adonis; R² = 0.43, *P* < 0.001; **Figure 1B**). PC1 and PC2 explain in total 83.2% of the variation in the data. Anisodine content of HQ (1.72 mg/g) was significantly higher than HH (0.82 mg/g) (*P* < 0.001), while anisodamine and atropine content of HH (anisodamine:0.77 mg/g, atropine: 3.67 mg/g) were significantly higher than HQ (anisodamine:0.36 mg/g, atropine: 1.45 mg/g) (*P* < 0.001) (**Table S2**). We also found a significant linear correlation between secondary metabolites and latitude; anisodine content had a significant positive correlation with latitude (*P* < 0.001), while anisodamine (*P* = 0.002) and atropine (*P* < 0.001) content had a significant negative correlation with latitude (**Figure 1D**). The dissimilarity of secondary metabolites showed a significant positive relationship with geographical distance (*P* < 0.001) (**Figure 1E**).

### Root-related microbiome of *A tanguticus*

**Figure 2:**
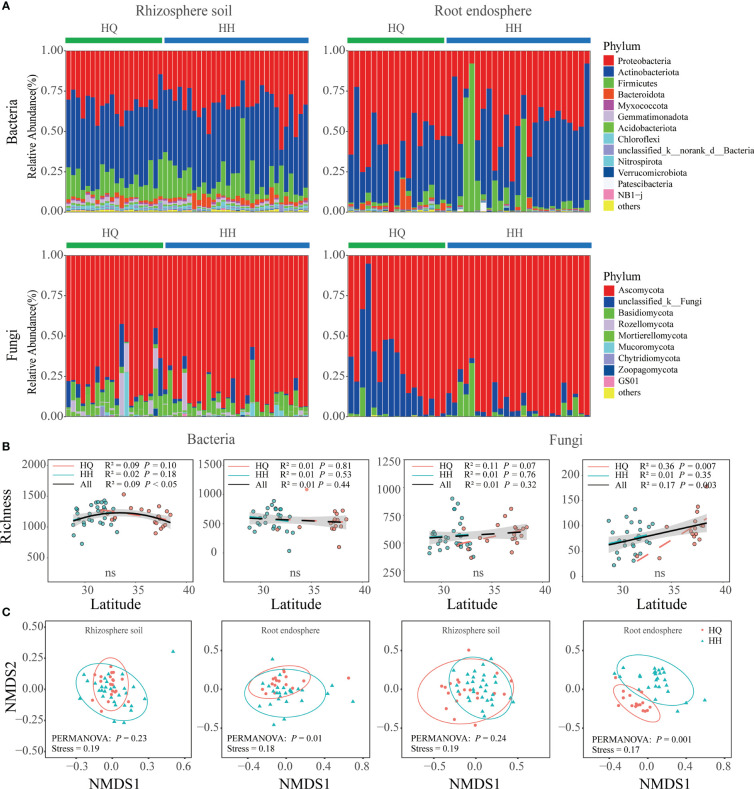
Microbial diversity and composition of *Anisodus tanguticus*. **(A)** Microbial composition at phylum level (relative abundance of > 0.1%). **(B)** Regression analysis of α-diversity and latitude. Differences between HQ and HH groups were determined using the Wilcox test. **(C)** Non-metric multidimensional scaling (NMDS) ordination plot based on Bray–Curtis dissimilarity.

The bacterial sequence in the rhizosphere soil and root endosphere consisted mainly of the phyla Actinobacteriota and Proteobacteria (**Figure 2A**). The three major phyla in the rhizosphere soil were Proteobacteria (33.55%), Actinobacteriota (45.01%), and Firmicutes (12.37%). The three major phyla in the root endosphere were Proteobacteria (48.89%), Actinobacteriota(39.03%), and Firmicutes (8.59%). Ascomycota was the most abundant fungal phyla in rhizosphere soil and root endosphere (**Figure 2A**). The three major phyla in the rhizosphere soil were Ascomycota (82.61%), Basidiomycota (7.21%), and unclassified_k:Fungi (3.64%). The three major phyla in the root endosphere were Ascomycota (82.48%), unclassified_k:Fungi (14.16%), and Glomeromycota (0.09%). The bacterial and fungal alpha diversity in rhizosphere soil and root endosphere all did not differ significantly between HQ and HH groups (**Figure 2B**). NMDS analysis results based on OTU levels showed a significant distinction in root endophytic bacterial and fungal communities (Adonis; bacteria: R² = 0.04, *P* = 0.01; fungi: R² = 0.09, *P* = 0.001), however, bacterial and fungal communities in rhizosphere soil were not significantly separated (**Figure 2C**). The distribution of endophytic fungi and bacteria appears to be geographically dependent.

**Figure 3:**
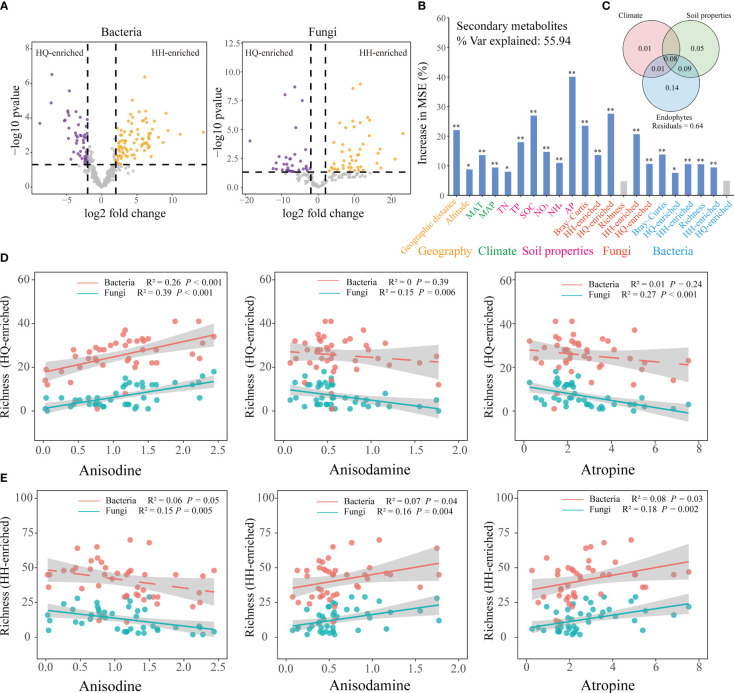
Effect of environmental factors on the tropane alkaloids content of *Anisodus tanguticus*. **(A)** edgeR is utilized to find differentially expressed OTU. **(B)** Random forest model showing the most important factors that impact on secondary metabolites. Higher MSE% values implied more important variables. Geographic variables include geographic distance and altitude; climate variables include MAT (mean annual temperature), MAP (mean annual precipitation); Soil properties include TN (soil total nitrogen), TP (soil total phosphorus), SOC (soil organic matter), NO_3_ (soil nitrate nitrogen), NH_4_ (soil ammonium nitrogen) and AP (soil available phosphorus); fungal diversity include Bray-Curtis (beta diversity), HH-enriched (HH-enriched OTUs beta diversity), HQ-enriched (HQ-enriched OTUs beta diversity), Richness (alpha diversity), HH-enriched (HH-enriched OTUs Richness), HQ-enriched (HQ-enriched OTUs Richness); bacterial diversity include Bray-Curtis (beta diversity), HH-enriched (HH-enriched OTUs beta diversity), HQ-enriched (HQ-enriched OTUs beta diversity), Richness (alpha diversity), HH-enriched (HH-enriched OTUs Richness), HQ-enriched (HQ-enriched OTUs Richness). **(C)** Variation partitioning analysis (VPA) differentiate the contributions of climate variables, soil chemical variables, and endophytes on secondary metabolites variations. Numbers indicate the proportion of explained variation and residuals indicate unexplained variations. **(D)** Regression analysis of richness of enriched endophytes in HQ group and three tropane alkaloids content. **(E)** Regression analysis of richness of enriched endophytes in HH group and three tropane alkaloids content.

EdgeR analysis was used to identify root bacterial and fungal OTUs that were differentially abundant between the HQ and HH groups (**Figure 3A**). EdgeR identified 49 bacterial OTUs differentially enriched in the HQ group belonged to Proteobacteria (26 OTUs), Acidobacteriota (13 OTUs), Bacteroidota (5 OTUs), Myxococcota (2 OTUs), Chloroflexi (1 OTUs), Gemmatimonadota (1 OTUs) and Spirochaetota (1 OTUs), and 86 OTUs enriched in the HH group belonged to Acidobacteriota (44 OTUs), Proteobacteria (29 OTUs), Firmicutes (11 OTUs), Bacteroidota (1 OTUs) and Patescibacteria (1 OTUs) (**Figure S1A**). EdgeR identified 39 fungal OTUs differentially enriched in the HQ group belonged to Ascomycota (18 OTUs), unclassified_k:Fungi (17 OTUs) and Basidiomycota (4 OTUs), and 46 OTUs enriched in the HH group belonged to Ascomycota (29 OTUs), unclassified_k:Fungi (6 OTUs) and Basidiomycota (11 OTUs) (**Figure S1B**).

### The main driver for the geographic distribution of tropane alkaloids

To further explore the main factors influencing the accumulation of tropane alkaloids, we selected climate, soil, and endophytes variables for further analysis. Variation partition analysis (VPA) was used to assess the contribution of climate, soil properties, and root endophytes to the geographic distribution of tropane alkaloids (**Figure 3C**). The root endophytes alone explained 14% of the variation, which was the largest contributor. Soil properties variables could independently explain 5% of the variation, and climate variables could explain 1% of the variation. These results show that endophytes were the main drivers contributing to the tropane alkaloids accumulation. Specifically, root endophytic fungi alone accounted for 11% of the observed variation, while root endophytic bacteria independently explained 5% of the variation (**Figure S2**). Endophytic fungi play a more significant role in the accumulation of tropane alkaloids compared to bacteria.

Random forests suggest that regionally enriched endophytic fungi have a greater impact on the accumulation of tropane alkaloids (**Figure 3B**). Mantel test also showed regionally enriched endophytic fungal beta diversity (All: R^2^ = -0.06, *P* = 0.80; HH-enriched: R^2^ = 0.26, *P* < 0.001; HQ-enriched: R^2^ = 0.26, *P* < 0.001) and alpha diversity (All: R^2^ = 0.14, *P* = 0.03; HH-enriched: R^2^ = 0.23, *P* < 0.001; HQ-enriched: R^2^ = 0.24, *P* < 0.001) were significantly correlated with secondary metabolites (**Table S3**). Richness of enriched endophytic fungi in HQ group has a significant (R^2^ = 0.39, *P* < 0.001) positive correlation with anisodine content, while have a significant negative relationship with anisodamine (R^2^ = 0.15, *P* = 0.006) and atropine (R^2^ = 0.27, *P* < 0.001) content (**Figure 3D**); richness of enriched endophytic fungi in HH group has a significant (R^2^ = 0.15, *P* = 0.005) negative correlation with anisodine content, while have a significant positive correlation with anisodamine (R^2^ = 0.16, *P* = 0.004) and atropine (R^2^ = 0.18, *P* = 0.002) content (**Figure 3E**). The correlation between the relative abundance of regionally enriched endophytic fungi and tropane alkaloids content is consistent with its relationship with richness (**Figure S3**). The presence of high levels of anisodine content in HQ showed a positive correlation with the abundance and diversity of enriched endophytic fungi in this region, while the presence of high levels of anisodamine and atropine content in HH showed a positive correlation with the abundance and diversity of enriched endophytic fungi in this region. Compared to endophytic fungi, the relative abundance of enriched endophytic bacteria shows a significant positive weak relationship with tropane alkaloids content (**Figure S4**).

### Relationship between tropane alkaloids accumulation and major root-related microbiome

**Figure 4:**
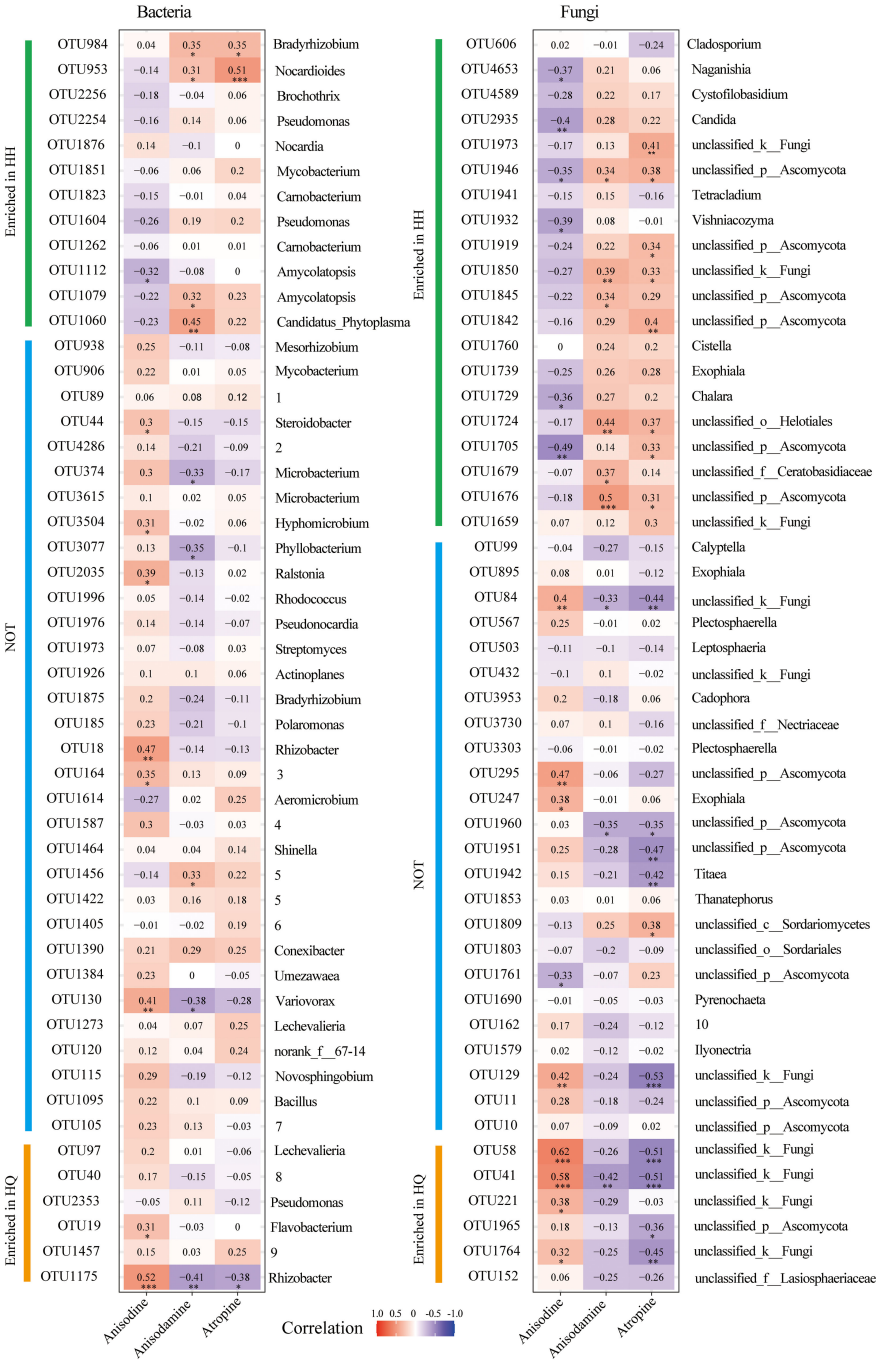
Spearman correlation analysis of dominant endophytes (the top 50 abundance) and differential tropane alkaloids of roots of *Anisodus tanguticus*. The numbers in the graph indicate the R-value. The colors in the graph indicate the P-value. The red and blue indicate positive and negative correlations, and the color depth indicates strong correlation (* P < 0.05, ** P < 0.01, *** P < 0.01). The English word indicates the genus to which the OTU belongs. Since some of the words are too long, we have put them in numbers, which are illustrated inside the legend. 1(unclassified_f:Micromonosporaceae), 2(unclassified_f:Microbacteriaceae), 3(norank_f:Rhizobiales_Incertae_Sedis), 4(Burkholderia-Caballeronia-Paraburkholderia), 5(Allorhizobium-Neorhizobium-Pararhizobium-Rhizobium), 6(unclassified_f:Xanthobacteraceae), 7(unclassified_f:Oxalobacteraceae), 8(unclassified_f:Rhodocyclaceae), 9(norank_f:norank_o:Microtrichales), 10(unclassified_f:Helotiales_fam_Incertae_sedis).

Spearman correlation analysis between major endophytic microorganisms and differential tropane alkaloids of *A. tanguticus* was performed. The top 50 most abundant bacterial OTUs were chosen, 12 of which enriched in the HH group and 6 of which enriched in the HQ group (**Figure 4A**). The top 50 most abundant fungal OTUs were chosen, 20 of which enriched in the HH group and 6 of which enriched in the HQ group (**Figure 4B**). For endophytic bacteria, anisodine was significant positive correlation with OTU18 (*Rhizobacter*), OTU19 (*Flavobacterium*), OTU130 (*Variovorax*), OTU164 (*norank_f:Rhizobiales_Incertae_Sedis*), OTU44 (*Steroidobacter*), OTU1175 (*Rhizobacter*), OTU2035 (*Ralstonia*), and OTU3504 (*Hyphomicrobium*) (**Figure 4A**). Among them, OTU19 and OTU1175 were enriched in the HQ group. Anisodamine was significant positive correlation with OTU953 (*Nocardioides*), OTU984 (*Bradyrhizobium*), OTU1060 (*Candidatus_Phytoplasma*), OTU1079 (*Amycolatopsis*), and OTU1456 (*Allorhizobium-Neorhizobium-Pararhizobium-Rhizobium*) (**Figure 4A**). Among them, OTU953, OTU984, OTU1060, and OTU1079 were enriched in the HH group. Atropine was significant positive correlation with OTU953 (*Nocardioides*) and OTU984 (*Bradyrhizobium*) (**Figure 4A**). Among them, OTU953 and OTU984 were enriched in the HH group. For endophytic fungi, anisodine was significant positive correlation with OTU41 (*unclassyfied_k_Fungi*), OTU58 (*unclassyfied_k_Fungi*), OTU84 (*unclassyfied_k_Fungi*), OTU129 (*unclassyfied_k_Fungi*), OTU221 (*unclassyfied_k_Fungi*), OTU247 (*Exophiala*), OTU295 (*unclassified_p_Ascomycota*), and OTU1764 (*unclassyfied_k_Fungi*) (**Figure 4B**). Among them, OTU41, OTU58, OTU221, and OTU1764 were enriched in the HQ group. Anisodamine was significant positive correlation with OTU1676 (*unclassified_p_Ascomycota*), OTU1679 (*unclassified_f_Ceratobasidiaceae*), OTU1724 (*unclassified_o_Helotiales*), OTU1845 (*unclassified_p_Ascomycota*), OTU1850 (*unclassyfied_k_Fungi*), and OTU1946 (*unclassified_p_Ascomycota*) (**Figure 4B**). They were all enriched in the HH group. Atropine was significant positive correlation with OTU1676 (*unclassified_p_Ascomycota*), OTU1705 (*unclassified_p_Ascomycota*), OTU1724 (*unclassified_o_Helotiales*), OTU1809 (*unclassified_c_Sordariomyc-etes*), OTU1842 (*unclassified_p_Ascomycota*), OTU1850 (*unclassyfied_k_Fungi*), OTU1919 (*unclassified_p_Ascomycota*), OTU1946 (*unclassified_p_Ascomycota*), and OTU1973 (*unclassyfied_k_Fungi*) (**Figure 4B**). Except for OTU 1809, all other OTUs were enriched in group HH. Although the 50 OTUs with the highest relative abundance were screened, the OTUs that were positively correlated with tropane alkaloid content were mainly microbial taxa enriched in different regions. The relative abundance of most unenriched OTUs showed no significant correlation with three alkaloids content.

### Enriched OTUs in the microbial co-occurrence network

**Figure 5:**
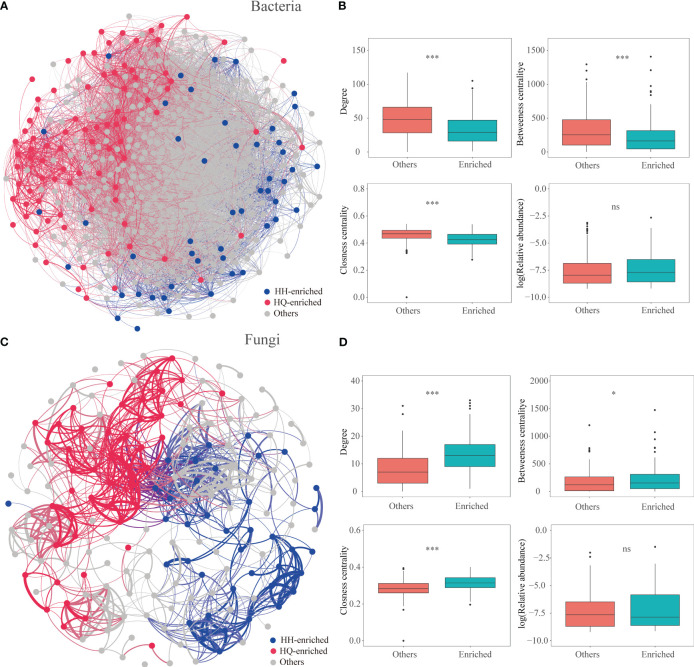
Co-occurrence patterns of the microbial community of *Anisodus tanguticus*. **(A, C)** Co-occurrence networks for bacterial and fungi communities based on Spearman’s correlations (r > |0.4|, FDR-corrected p < 0.05). The red and blue dots represent OTUs which enriched in HQ and HH, respectively. **(B, D)** Node-level topological features of HQ- and HH-enriched OTUs. Differences were determined using the Wilcox test. The significance levels are as follows: P < 0.05, one asterisk (*); P < 0.001, three asterisks (***).

In our study, the results showed that regionally enriched endophytes, especially endophytic fungi, are more important for the accumulation of tropane alkaloids. Previous studies have shown that microbial taxa with crucial functions are often closely related to each other (Fan et al., 2021). Co-occurrence networks have been confirmed as an effective tool to study the species interactions of complex microbial communities (Faust and Raes, 2012; Zhang et al., 2022a). In our study, we constructed microbial co-occurrence networks based on correlation relationships (**Figure 5**; **Table S4**). The root bacterial network consisted of 10485 associations among 473 OTUs, and the fungal network consisted of 956 associations among 178 OTUs. For endophytic bacteria, 49 and 86 significantly enriched bacterial OTUs were identified in the HQ and HH groups, respectively. OTUs enriched in different regions are scattered in the bacterial network (**Figure 5A**). In terms of the network-level topological features, values of degree (enriched bacterial OTUs: 33.63, other OTUs: 48.61, *P* < 0.001), betweenness centrality (enriched bacterial OTUs: 225.49, other OTUs: 315.96, *P* < 0.001), and closeness centrality (enriched bacterial OTUs: 0.43, other OTUs: 0.46, *P* < 0.001) were significantly lower in enriched OTUs than others (except the enriched OTUs) (**Figure 5B**). For endophytic fungi, 39 and 46 significantly enriched fungal OTUs were identified in the HQ and HH groups, respectively. OTUs enriched in different regions formed independent modules (**Figure 5C**). Values of the degree (enriched fungal OTUs: 13.66, other OTUs: 8.08, *P* < 0.001), betweenness centrality (enriched fungal OTUs: 229.86, other OTUs: 182.58, *P* < 0.05), and closeness centrality (enriched fungal OTUs: 0.32, other OTUs: 0.28, *P* < 0.001) were significantly higher in enriched OTUs than others (**Figure 5D**), indicating that fungal enriched OTUs were more interconnected.

## Discussion

As an influential factor impacting the quality of medicinal plants, ecological factors have consistently received significant attention in research. In recent years, numerous studies have discovered that climate factors, soil nutrients (Dar et al., 2016), heavy metals (Ma et al., 2018), and altitude can influence the accumulation of secondary metabolites in plants. Chen et al. demonstrated a significant difference in the chemical composition of *A. tanguticus* samples between the Tibetan and non-Tibet regions (Chen et al., 2022b). This disparity is attributed to the distinct geographical factors of high altitude, low temperatures, and low precipitation in the Tibetan region. However, these studies primarily concentrate on abiotic factors and lack investigation on biotic factors, such as plant microbiomes. In our study, we examined the impact of soil properties, climate variables, and the plant microbiome on the accumulation of tropane alkaloids in the roots of *A. tanguticus*. Our results also show that the Hengduan Mountains divides the *A. tanguticus* samples into two significantly different parts, which is consistent with their findings. However, in addition to the differences in environmental factors, we also found that endophytes, especially endophytic fungi, were also divided into two significantly different parts. Subsequent analysis demonstrated that endophytes was the largest contributor for the accumulation of tropane alkaloids, followed by soil properties and climate variables. Our study, therefore, complements previous work in this area very well.

In our study, richness and relative abundance of enriched endophytic fungi in HQ group has a significant positive correlation with anisodine content, while richness and relative abundance of enriched endophytic fungi in HH group has a significant positive correlation with anisodamine and atropine content. In previous study, there was a significant correlation between dominant bacteria in the root and differential metabolites in *Ephedra sinica* (Miao et al., 2022) and *Panax quinquefolius* (Li et al., 2023). There is also a number of studies showing that endophytic fungi were significantly related to differential metabolites in *Cynomorium songaricum* (Cui et al., 2019) and *Rheum palmatum* (Chen et al., 2021). We speculate that endophytes have an impact on the biosynthetic pathways of tropane alkaloids. Endophytic Fungus Fusarium oxysporum isolated from *Catharanthus roseus* can produce vinblastine and vincristine (Das et al., 2020). Endophytic Fungus *Fusarium solani* isolated from *Fusarium solani* can produce camptothecin (Ran et al., 2017). Endophytic Fungus *Neotyphodium coenophialum* isolated from *Festuca arundinacea* can produce ergot alkaloids. We have identified certain microbial taxa that are closely associated with tropane alkaloids accumulation (such as *Rhizobacter*, *Flavobacterium*, *Variovorax*, *Ralstonia*, *Rhizobiales_Incertae_Sedis*, *Bradyrhizobium*, *Steroidobacter*, *Hyphomicrobium*, *Candidatus_Phytoplasma*, *Amycolatopsis*, *Nocardioides*, *unclassyfied_k_Fungi* and *unclassified_p_Ascomycota*), but it is currently unclear whether their involvement in the metabolic process is direct or indirect. But we found that some of these taxa are closely related to plant nitrogen fixation, such as genus *Rhizobacter*, *Rhizobiales_Incertae_Sedis*, *Bradyrhizobium*, *Steroidobacter*, *Hyphomicrobium*, and *Nocardioides* (Mezzasalma et al., 2018; Ulzen et al., 2018). As a class of nitrogenous compounds, nitrogen supplementation significantly increased the content of tropane alkaloids (Lucini et al., 2020). However, it is regrettable that most endophytic fungi which significantly associated with the content of the three alkaloids could not be classified which suggested that there are currently no reports of these fungal OTU types. Currently, only approximately 10 000 have been studied and classified in approximately 5.1 million fungal species (Blackwell, 2011). We believe that, with the development of sequencing technology, more and more fungal species will be detected which is very important for subsequent research (Kaul et al., 2016).

However, the unique ecological environment of Tibetan areas cannot be ignored. The unique high salt soil environment of *Citrus reticulata* core production area breeds unique microbial taxa that promote the accumulation of monoterpene (Su et al., 2023). Differences in plant microbiomes are often the result of environmental factors. A study conducted on multiple plant phyla indicates that soil type significantly affects the composition of root-associated bacterial communities (Yeoh et al., 2017). This is primarily due to soil microorganisms serving as the primary source of plant endophytes. This difference is often reflected at the genus level, at the phylum level they are similar (U'Ren et al., 2012). In our study, the microbial taxa that were significantly positively correlated with anisodine, anisodamine, and atropine differed at the genus level. The unique climate and soil conditions in Tibetan areas have shaped unique microorganisms that may affect the synthesis of tropane alkaloids. However, research has also demonstrated that the migration of soil microbes to the root system is influenced by the host and that host genotypes also play a role in selecting plant microbes (Wagner et al., 2016). The eastern Himalayas–Hengduan Mountains region, one of the world’s most biodiverse regions, also serves as a refuge for numerous plant species. During the Quaternary Ice Age, plants in the plateau sought refuge in the southeastern plateaus. After the end of the Ice Age, part of the *A. tanguticus* population located on the edge of the eastern Himalayas mountain range may have migrated westward, while part of the *A. tanguticus* population located on the edge of the Hengduan Mountains range may have migrated northward (Xing and Ree, 2017; Wan et al., 2016). Different genetic patterns are formed due to different climatic conditions and high mountains barrier (Chen et al., 2019). Differences in genotypes cause plants to recruit different microbial taxa, which may affect the synthesis of secondary metabolites, resulting in geographical differences (Shenton et al., 2016).

The synthesis of secondary metabolites of medicinal plants is a multi-step synthesis process involving endophytes. Every endophyte has the distinct capability to upregulate or downregulate specific genes that are involved in regulating the biosynthesis of secondary metabolites (Ray et al., 2019). It is impossible for an single endophyte to upregulate all biosynthetic steps, limiting the realization of maximum yield. Combined inoculation with SM1B (*Acinetobacter* sp.) and SM3B (*Marmoricola* sp.), which were isolated from capsule of alkaloid rich Sampada, significantly increased morphine content in *Papaver somniferum* compared with inoculation alone (Ray et al., 2019). Therefore, through a combination of multiple endophytes, the deficiencies of the endophytes are supplemented to achieve maximum production. Microorganisms involved in the synthesis of secondary metabolites and closely related to each other are the best candidates. In agroecosystems, the biodiversity of highly interconnected taxa in soils was significantly positively correlated with crop yield (Fan et al., 2021). In our study, endophytic fungi that were significantly positively correlated with secondary metabolite synthesis had high interconnectivity, which is consistent with previous findings. This further proves that highly related microorganisms have more important ecological functions. Isolating and cultivating these microbial taxa to produce microbial fertilizers, or for use in industrial production, can maximize yields and desired products.

## Conclusion

Using multi-omics approaches, we investigated the effects of soil conditions, climatic factors, and endophytes on the production of tropane alkaloids in *A. tanguticus* roots. We observed that tropane alkaloids were geography dependent and root endophytes are the primary drivers of tropane alkaloids accumulation, especially endophytic fungi. The alpha diversity and relative abundance of enriched endophytes in different regions promote different tropane alkaloids accumulation which results in different alkaloids with different biogeographic patterns. These microbial compositions exhibit variations at the genus level, but they remain functionally conserved, with a dominant presence of biological nitrogen fixation capacity. This study further confirmed that endophytes are closely related to tropane alkaloids accumulation of *A. tanguticus* and provides strong support for the study of the authenticity of medicinal herbs.

## Data availability statement

The datasets presented in this study can be found in online repositories. The names of the repository/repositories and accession number(s) can be found in the article/**Supplementary Material**.

## Author contributions

BW: Conceptualization, Investigation, Software, Validation, Visualization, Writing – original draft, Writing – review & editing. CC: Investigation, Methodology, Validation, Writing – review & editing. YX: Writing – review & editing. YH: Investigation, Writing – review & editing. YG: Investigation, Writing – review & editing. ZK: Investigation, Writing – review & editing. XW: Investigation, Writing – review & editing. YD: Investigation, Writing – review & editing. SF: Investigation, Writing – review & editing. GZ: Conceptualization, Funding acquisition, Investigation, Validation, Writing – review & editing.

## References

[B1] Archer E. (2022). rfPermute: Estimate permutation p-Values for random forest importance metrics. R package version 2.5.1. Available at: https://CRAN.R-project.org/package=rfPermute.

[B2] Bao S. D. (2018). Soil and Agricultural Chemistry Analysis. 3rd Ed (Beijing: China Agriculture Press).

[B3] Blackwell M. (2011). The fungi: 1, 2, 3 ... 5.1 million species? Am. J. Bot. 98, 426–438. doi: 10.3732/ajb.1000298 21613136

[B4] Chen J. H. Huang Y. Brachi B. Yun Q. Z. Zhang W. Lu W.. (2019). Genome-wide analysis of Cushion willow provides insights into alpine plant divergence in a biodiversity hotspot. Nat. Commun. 10, 5230. doi: 10.1038/s41467-019-13128-y 31745089 PMC6864086

[B5] Chen D. W. Jia L. Y. Hou Q. Z. Zhao X. Sun K. (2021). Analysis of endophyte diversity of rheum palmatum from different production areas in gansu province of China and the association with secondary metabolite. Microorganisms 9, 978. doi: 10.3390/microorganisms9050978 33946518 PMC8147242

[B6] Chen C. Li J. J. Xiong F. Wang B. Xiao Y. M. Zhou G. Y. (2022a). Multivariate statistical analysis of tropane alkaloids in Anisodus tanguticus (Maxim.) Pascher from different regions to trace geographical origins. Acta Chromatogr. 34, 422–429. doi: 10.1556/1326.2021.00952

[B7] Chen C. Wang B. Li J. J. Xiong F. Zhou G. Y. (2022b). Multivariate statistical analysis of metabolites in anisodus tanguticus (Maxim.) pascher to determine geographical origins and network pharmacology. Front. Plant Sci. 13, 927336. doi: 10.3389/fpls.2022.927336 35845631 PMC9277180

[B8] Cui J. L. Gong Y. Vijayakurnar V. Zhang G. Wang M. L. Wang J. H.. (2019). Correlation in chemical metabolome and endophytic mycobiome in cynomorium songaricum from different desert locations in China. J. Agr. Food Chem. 67, 3554–3564. doi: 10.1021/acs.jafc.9b00467 30860831

[B9] Dar T. A. Uddin M. Khan M. M. A. Ali A. Varshney L. (2016). Modulation of alkaloid content, growth and productivity of L. using irradiated sodium alginate in combination with soil applied phosphorus. J. Appl. Res. Med. Aromat. Plants. 3, 200–210. doi: 10.1016/j.jarmap.2016.05.003

[B10] Das A. Sarkar S. Bhattacharyya S. Gantait S. (2020). Biotechnological advancements in Catharanthus roseus (L.) G. Don. Appl. Microbiol. Biot. 104, 4811–4835. doi: 10.1007/s00253-020-10592-1 32303816

[B11] Fan K. K. Delgado-Baquerizo M. Guo X. S. Wang D. Z. Zhu Y. G. Chu H. Y. (2021). Biodiversity of key-stone phylotypes determines crop production in a 4-decade fertilization experiment. Isme. J. 15, 550–561. doi: 10.1038/s41396-020-00796-8 33028975 PMC8027226

[B12] Fan B. S. Zhang E. H. Wu M. Guo J. M. Su D. F. Liu X.. (2016). Activation of alpha 7 Nicotinic Acetylcholine Receptor Decreases On-site Mortality in Crush Syndrome through Insulin Signaling-Na/K-ATPase Pathway. Front. Pharmacol. 7, 79. doi: 10.3389/fphar.2016.00079 27065867 PMC4810156

[B13] Faust K. Raes J. (2012). Microbial interactions: from networks to models. Nat. Rev. Microbiol. 10, 538–550. doi: 10.1038/nrmicro2832 22796884

[B14] Frank E. Harrell J. R. (2022). Hmisc: Harrell Miscellaneous. R package version 4.7-2. Available at: https://CRAN.R-project.org/package=Hmisc.

[B15] Griffin W. J. Lin G. D. (2000). Chemotaxonomy and geographical distribution of tropane alkaloids. Phytochemistry 53, 623–637. doi: 10.1016/s0031-9422(99)00475-6 10746874

[B16] Hu L. S. Xu Z. P. Wang M. J. Fan R. Yuan D. J. Wu B. D.. (2019). The chromosome-scale reference genome of black pepper provides insight into piperine biosynthesis. Nat. Commun. 10, 4702. doi: 10.1038/s41467-019-12607-6 31619678 PMC6795880

[B17] Kaul S. Sharma T. Dhar M. K. (2016). "Omics" Tools for better understanding the plant-endophyte interactions. Front. Plant Sci. 7. doi: 10.3389/fpls.2016.00955 PMC4925718 27446181

[B18] Kohnen-Johannsen K. L. Kayser O. (2019). Tropane alkaloids: chemistry, pharmacology, biosynthesis and production. Molecules 24, 796. doi: 10.3390/molecules24040796 30813289 PMC6412926

[B19] Li R. Duan W. Y. Ran Z. F. Chen X. L. Yu H. X. Fang L.. (2023). Diversity and correlation analysis of endophytes and metabolites of L. @ in various tissues. BMC Plant Biol. 23, 275. doi: 10.1186/s12870-023-04340-6 37226095 PMC10210350

[B20] Li L. Li Z. M. Wang Y. Z. (2022). A method of two-dimensional correlation spectroscopy combined with residual neural network for comparison and differentiation of medicinal plants raw materials superior to traditional machine learning: a case study on Eucommia ulmoides leaves. Plant Methods 18, 102. doi: 10.1186/s13007-022-00935-6 35964064 PMC9375363

[B21] Li J. Zhao G. Z. Varma A. Qin S. Xiong Z. Huang H. Y.. (2012). An endophytic pseudonocardia species induces the production of artemisinin in artemisia annua. PloS One 7, e51410. doi: 10.1371/journal.pone.0051410 23251523 PMC3520919

[B22] Liu J. Abdelfattah A. Norelli J. Burchard E. Schena L. Droby S.. (2018). Apple endophytic microbiota of different rootstock/scion combinations suggests a genotype-specific influence. Microbiome 6, 18. doi: 10.1186/s40168-018-0403-x 29374490 PMC5787276

[B23] Liu Y. Li D. Gao H. Li Y. H. Chen W. M. Jiao S.. (2022). Regulation of soil micro-foodwebs to root secondary metabolites in cultivated and wild licorice plants. Sci. Total. Environ. 828, 154302. doi: 10.1016/j.scitotenv.2022.154302 35276159

[B24] Liu Z. C. Wang Y. Liu Y. M. (2019b). Geographical origins and varieties identification of hops (Humulus lupulus L.) by multi-metal elements fingerprinting and the relationships with functional ingredients. Food Chem. 289, 522–530. doi: 10.1016/j.foodchem.2019.03.099 30955644

[B25] Liu Y. Wang H. Peng Z. H. Li D. Chen W. M. Jiao S.. (2021). Regulation of root secondary metabolites by partial root-associated microbiotas under the shaping of licorice ecotypic differentiation in northwest China. J. Integr. Plant Biol. 63, 2093–2109. doi: 10.1111/jipb.13179 34655272

[B26] Liu X. Xia B. Yu H. K. Hu L. Z. Fan S. M. Xiao D.. (2019a). Atropine premedication facilitates ultrasound-guided reduction by saline enema in children with intussusception. Front. Pharmacol. 10, 862. doi: 10.3389/fphar.2019.00862 31427971 PMC6688091

[B27] Lockery J. E. Broder J. C. Ryan J. Stewart A. C. Woods R. L. Chong T. T. J.. (2021). A cohort study of anticholinergic medication burden and incident dementia and stroke in older adults. J. Gen. Intern. Med. 36, 1629–1637. doi: 10.1007/s11606-020-06550-2 33754317 PMC8175463

[B28] Lucini L. Miras-Moreno B. Rouphael Y. Cardarelli M. Colla G. (2020). Combining molecular weight fractionation and metabolomics to elucidate the bioactivity of vegetal protein hydrolysates in tomato plants. Front. Plant Sci. 11, 976. doi: 10.3389/fpls.2020.00976 32695133 PMC7338714

[B29] Luo J. Q. Zhang Z. C. Hou Y. Z. Diao F. W. Hao B. H. Bao Z. H.. (2021). Exploring microbial resource of different rhizocompartments of dominant plants along the salinity gradient around the hypersaline lake ejinur. Front. Microbiol. 12, 698479. doi: 10.3389/fmicb.2021.698479 34322109 PMC8312270

[B30] Ma S. Zhu G. Yu F. Zhu G. Wang D. Wang W.. (2018). Effects of manganese on accumulation of Glycyrrhizic acid based on material ingredients distribution of Glycyrrhiza uralensis. Ind. Crops Prod. 112, 151–159. doi: 10.1016/j.indcrop.2017.09.035

[B31] Maehara S. Agusta A. Tokunaga Y. Shibuya H. Hata. T. (2019). Endophyte composition and Cinchona alkaloid production abilities of Cinchona ledgeriana cultivated in Japan. J. Nat. Med-Tokyo. 73, 431–438. doi: 10.1007/s11418-018-1273-z 30552602

[B32] Mezzasalma V. Sandionigi A. Guzzetti L. Galimberti A. Grando M. S. Tardaguila J.. (2018). Geographical and cultivar features differentiate grape microbiota in northern Italy and Spain vineyards. Front. Microbiol. 9, 946. doi: 10.3389/fmicb.2018.00946 29867854 PMC5962658

[B33] Miao S. M. Xia Y. Cui J. L. Wang J. H. Wang M. L. (2022). Correlation analysis between differential metabolites and bacterial endophytes of Ephedra sinica in different years. Ind. Crops Prod. 175, 114250. doi: 10.1016/j.indcrop.2021.114250

[B34] Oksanen J. Kindt R. Legendre P. O'Hara R. B. (2011). Vegan: community ecology package. R package version 2.0–2. Available at: https://CRAN.R-project.org/package=vegan.

[B35] Ran X. Q. Zhang G. Li S. Wang J. F. (2017). Characterization and antitumor activity of camptothecin from endophytic fungus Fusarium solani isolated from Camptotheca acuminate. Afr. Health Sci. 17, 566–574. doi: 10.4314/ahs.v17i2.34 29062355 PMC5637045

[B36] Ray T. Pandey S. S. Pandey A. Srivastava M. Shanker K. Kalra A. (2019). Endophytic consortium with diverse gene-regulating capabilities of benzylisoquinoline alkaloids biosynthetic pathway can enhance endogenous morphine biosynthesis in papaver somniferum. Front. Microbiol. 10, 925. doi: 10.3389/fmicb.2019.00925 31114562 PMC6503101

[B37] R Core Team (2021). R: A language and environment for statistical computing (Vienna, Austria: R Foundation for Statistical Computing). Available at: https://www.R-project.org/.

[B38] Robinson M. D. McCarthy D. J. Smyth G. K. (2010). edgeR: a Bioconductor package for differential expression analysis of digital gene expression data. Bioinformatics 26, 139–140. doi: 10.1093/bioinformatics/btp616 19910308 PMC2796818

[B39] Schulz B. Boyle C. (2005). The endophytic continuum. Mycological. Res. 109, 661–686. doi: 10.1017/S095375620500273x 16080390

[B40] Shenton M. Iwamoto C. Kurata N. Ikeo K. (2016). Effect of wild and cultivated rice genotypes on rhizosphere bacterial community composition. Rice 9, 42. doi: 10.1186/s12284-016-0111-8 27557607 PMC4996804

[B41] Shim K. H. Kang M. J. Sharma N. An S. S. A. (2022). Beauty of the beast: anticholinergic tropane alkaloids in therapeutics. Nat. Prod. Bioprospect. 12, 33. doi: 10.1007/s13659-022-00357-w 36109439 PMC9478010

[B42] Stierle A. Strobel G. Stierle D. (1993). Taxol and taxane production by taxomyces-andreanae, an endophytic fungus of pacific yew. Science 260, 214–216. doi: 10.1126/science.8097061 8097061

[B43] Su J. M. Wang Y. Y. Bai M. Peng T. H. Li H. S. Xu H. J.. (2023). Soil conditions and the plant microbiome boost the accumulation of monoterpenes in the fruit of Citrus reticulata `Chachi'. Microbiome 11, 61. doi: 10.1186/s40168-023-01504-2 36973820 PMC10044787

[B44] Tian T. Wang Y. J. Huang J. P. Li J. Xu B. Y. Chen Y.. (2022). Catalytic innovation underlies independent recruitment of polyketide synthases in cocaine and hyoscyamine biosynthesis. Nat. Commun. 13, 4994. doi: 10.1038/s41467-022-32776-1 36008484 PMC9411544

[B45] U'Ren J. M. Lutzoni F. Miadlikowska J. Laetsch A. D. Arnold A. E. (2012). Host and geographic structure of endophytic and endolichenic fungi at a continental scale. Am. J. Bot. 99, 898–914. doi: 10.3732/ajb.1100459 22539507

[B46] Ulzen J. Abaidoo R. C. Ewusi-Mensah N. Masso C. (2018). On-farm evaluation and determination of sources of variability of soybean response to Bradyrhizobium inoculation and phosphorus fertilizer in northern Ghana. Agr. Ecosyst. Environ. 267, 23–32. doi: 10.1016/j.agee.2018.08.007 30449913 PMC6167739

[B47] Wagner M. R. Lundberg D. S. del Rio T. G. Tringe S. G. Dangl J. L. Mitchell-Olds T. (2016). Host genotype and age shape the leaf and root microbiomes of a wild perennial plant. Nat. Commun. 7, 12151. doi: 10.1038/ncomms1215 27402057 PMC4945892

[B48] Wan D. S. Feng J. J. Jiang D. C. Mao K. S. Duan Y. W. Miehe G.. (2016). The Quaternary evolutionary history, potential distribution dynamics, and conservation implications for a Qinghai-Tibet Plateau endemic herbaceous perennial, Anisodus tanguticus (Solanaceae). Ecol. Evol. 6, 1977–1995. doi: 10.1002/ece3.2019 27099706 PMC4831433

[B49] Wang B. Chen C. Xiao Y. Chen K. Wang J. Zhou G. Y. (2023). Temperature thresholds drive the biogeographic pattern of root endophytic fungal diversity in the Qinghai-Tibet Plateau. Sci. Total. Environ. 889, 164270. doi: 10.1016/j.scitotenv.2023.164270 37211120

[B50] Wickham H. (2016). ggplot2: Elegant graphics for data analysis (New York: Springer-Verlag).

[B51] Xing Y. W. Ree R. H. (2017). Uplift-driven diversification in the Hengduan Mountains, a temperate biodiversity hotspot. Proc. Natl. Acad. Sci. U.S.A. 114, E3444–E3451. doi: 10.1073/pnas.1616063114 28373546 PMC5410793

[B52] Xu L. L. Han T. Wu J. Z. Zhang Q. Y. Zhang H. Huang B. K.. (2009). Comparative research of chemical constituents, antifungal and antitumor properties of ether extracts of Panax ginseng and its endophytic fungus. Phytomedicine 16, 609–616. doi: 10.1016/j.phymed.2009.03.014 19403293

[B53] Xu Q. Zhao X. (2017). Progress in clinical application of compound anisodine injection on ophthalmology. Chin. J. Clin. Pharmacol. 33, 861–864. doi: 10.13699/j.cnki.1001-6821.2017.09.025

[B54] Yeoh Y. K. Dennis P. G. Paungfoo-Lonhienne C. Weber L. Brackin R. Ragan M. A.. (2017). Evolutionary conservation of a core root microbiome across plant phyla along a tropical soil chronosequence. Nat. Commun. 8, 215. doi: 10.1038/s41467-017-00262-8 28790312 PMC5548757

[B55] Yu J. T. Zhang X. N. Xu C. Y. Hao M. H. Choe C. He H. J. (2022). Thinning can increase shrub diversity and decrease herb diversity by regulating light and soil environments. Front. Plant Sci. 13, 948648. doi: 10.3389/fpls.2022.948648 35991461 PMC9389291

[B56] Yu Y. T. Zhu C. Hong Y. C. Chen L. Huang Z. P. Zhou J. C.. (2021). Effectiveness of anisodamine for the treatment of critically ill patients with septic shock: a multicentre randomized controlled trial. Crit. Care 25, 349. doi: 10.1186/s13054-021-03774-4 34579741 PMC8474812

[B57] Zhai X. Jia M. Chen L. Zheng C. J. Rahman K. Han T.. (2017). The regulatory mechanism of fungal elicitor-induced secondary metabolite biosynthesis in medical plants. Crit. Rev. Microbiol. 43, 238–261. doi: 10.1080/1040841x.2016.1201041 27936989

[B58] Zhang H. J. Yan Y. Lin T. H. Xie W. J. Hu J. Hou F. R.. (2022a). Disentangling the mechanisms shaping the prokaryotic communities in a eutrophic bay. Microbiol. Spectr. 10, 2165–0497. doi: 10.1128/spectrum.01481-22 PMC9241920 35638815

[B59] Zhang L. Y. Zhang M. L. Huang S. Y. Li L. J. Gao Q. Wang Y.. (2022b). A highly conserved core bacterial microbiota with nitrogen-fixation capacity inhabits the xylem sap in maize plants. Nat. Commun. 13, 3361. doi: 10.1038/s41467-022-31113-w 35688828 PMC9187771

[B60] ZhouW.WangC.HaoX.ChenF.HuangQ.LiuT.. (2023). A chromosome-level genome assembly of anesthetic drug-producing Anisodus acutangulus provides insights into its evolution and the biosynthesis of tropane alkaloids. Plant Commun. 5, 100680. doi: 10.1016/j.xplc.2023.100680 37660252

