# Supplementary material for: Geographically associated endophytic fungi contribute to the tropane alkaloids accumulation of Anisodus tanguticus
Source: Front Plant Sci. 2023 Nov 30;14:1297546. doi: 10.3389/fpls.2023.1297546 (PMC10720625; doi:10.3389/fpls.2023.1297546)
Supplement: Supplementary file 1 [file DataSheet_1.docx]

**Geographically associated endophytes contribute to the** **secondary metabolite accumulation** **of** ***Anisodus tanguticus***

Bo Wang^1,2^ Chen Chen^1^, Yuanming Xiao^1^, Yan He^3^, Ying Gao^3^, Zongxiu Kang^3^, Xiaoxuan Wei^3^, Yujie Deng^3^, Shihong Feng^4^ and Guoying Zhou^1,*^

1 CAS Key Laboratory of Tibetan Medicine Research, Northwest Institute of Plateau Biology, China

2 University of Chinese Academy of Sciences, Beijing, China

3 Datong Beichuan Heyuan District National Nature Reserve, Xining, China

4 Chengdu Tianxianzi agricultural science and technology development Co., LTD, Chengdu, China

*Corresponding author: Dr. Guoying Zhou, Tel: +86-971-6159630, Fax: +86-971-

6143282, E-mail: zhougy@nwipb.cas.cn

ORCID: https://orcid.org/0000-0003-2485-6172

Address: 23# Xinning Road, Xining, Qinghai, P. R. China

Table S1 Summary of the locations and climatic characteristics of the selected sampling sites

| ID | sample site | lon | lat | Altitude(m) | MAT(℃) | MAP (mm) |
| --- | --- | --- | --- | --- | --- | --- |
| HH1 | X_2 | 97.3655 | 33.1585 | 3926 | -0.08 | 524 |
| HH2 | X_3 | 96.9933 | 32.8528 | 3905 | 2.55 | 510 |
| HH3 | X_4 | 96.6419 | 32.7982 | 4174 | -0.50 | 556 |
| HH4 | X_6 | 96.5187 | 32.5784 | 4003 | 1.98 | 530 |
| HH5 | X_7 | 96.4902 | 32.0407 | 3771 | 3.60 | 533 |
| HH6 | X_52 | 98.7630 | 31.9603 | 3611 | 3.13 | 584 |
| HH7 | X_8 | 96.4394 | 31.9487 | 4318 | 0.93 | 574 |
| HH8 | X_38 | 93.8452 | 31.9125 | 4061 | 1.73 | 570 |
| HH9 | X_53 | 99.3301 | 31.8647 | 3744 | 2.29 | 608 |
| HH10 | X_37 | 93.5733 | 31.7959 | 4246 | -0.66 | 601 |
| HH11 | X_34 | 92.7466 | 31.7469 | 4253 | 0.49 | 493 |
| HH12 | X_41 | 94.7791 | 31.6394 | 4087 | 1.57 | 616 |
| HH13 | X_9 | 96.3419 | 31.5013 | 4115 | 1.94 | 578 |
| HH14 | X_46 | 97.4240 | 31.4708 | 3594 | 4.26 | 531 |
| HH15 | X_42 | 95.4877 | 31.4521 | 4097 | 1.96 | 599 |
| HH16 | X_50 | 97.9541 | 31.3044 | 3932 | 3.31 | 557 |
| HH17 | X_48 | 97.5957 | 31.2903 | 4097 | 1.70 | 564 |
| HH18 | X_45 | 96.5067 | 31.2281 | 3877 | 3.50 | 574 |
| HH19 | X_10 | 96.8486 | 31.1106 | 3857 | 3.46 | 554 |
| HH20 | X_11 | 96.9983 | 31.0953 | 3996 | 2.53 | 563 |
| HH21 | X_12 | 97.1950 | 30.6722 | 4497 | 0.38 | 584 |
| HH22 | X_14 | 97.0587 | 30.6143 | 4324 | 1.40 | 585 |
| HH23 | X_15 | 97.1696 | 30.4559 | 4270 | 1.82 | 584 |
| HH24 | X_31 | 91.0258 | 30.4214 | 4257 | 1.95 | 416 |
| HH25 | X_30 | 90.7169 | 29.9169 | 3945 | 4.46 | 410 |
| HH26 | X_18 | 92.6257 | 29.8716 | 4066 | 1.95 | 436 |
| HH27 | X_27 | 89.2083 | 29.6952 | 4071 | 3.80 | 392 |
| HH28 | X_29 | 90.7204 | 29.3916 | 3694 | 7.01 | 400 |
| HH29 | X_28 | 89.8215 | 29.2600 | 3862 | 5.60 | 345 |
| HH30 | X_23 | 92.3401 | 29.2289 | 3652 | 5.27 | 344 |
| HH31 | X_24 | 90.4779 | 29.1474 | 4573 | 1.99 | 309 |
| HH32 | X_22 | 91.9551 | 28.8847 | 4288 | 1.67 | 301 |
| HQ1 | Q_22 | 99.5909 | 38.4232 | 3245 | -1.70 | 376 |
| HQ2 | Q_26 | 100.4497 | 38.0719 | 3092 | -0.32 | 409 |
| HQ3 | Q_20 | 101.8089 | 37.9068 | 2577 | 1.75 | 381 |
| HQ4 | Q_19 | 101.9215 | 37.7119 | 2643 | 0.37 | 429 |
| HQ5 | Q_30 | 101.4033 | 37.5598 | 3118 | -0.24 | 473 |
| HQ6 | Q_9 | 102.2585 | 37.4843 | 3013 | -0.70 | 450 |
| HQ7 | Q_16 | 101.8998 | 37.3794 | 3133 | -0.29 | 482 |
| HQ8 | Q_6 | 102.2566 | 37.2990 | 3025 | 0.17 | 443 |
| HQ9 | Q_5 | 102.2631 | 37.2836 | 2925 | 0.15 | 443 |
| HQ10 | Q_29 | 101.5433 | 37.2252 | 3006 | -0.55 | 502 |
| HQ11 | Q_11 | 102.8013 | 37.1942 | 2877 | 1.02 | 393 |
| HQ12 | Q_2 | 102.2507 | 37.0299 | 3061 | 0.54 | 458 |
| HQ13 | Q_14 | 102.6846 | 36.9618 | 2722 | 2.10 | 394 |
| HQ14 | Q_1 | 102.1009 | 36.8949 | 2901 | 1.51 | 456 |
| HQ15 | X_65 | 101.5430 | 36.2970 | 3111 | 1.73 | 487 |
| HQ16 | X_64 | 101.0825 | 35.5724 | 3411 | 1.25 | 482 |
| HQ17 | X_62 | 100.4380 | 34.5269 | 3721 | -1.13 | 545 |
| HQ18 | X_61 | 100.1548 | 33.8211 | 3897 | -1.01 | 592 |
| HQ19 | X_58 | 100.7254 | 32.9557 | 3584 | 2.06 | 714 |
| HQ20 | X_56 | 100.5246 | 32.6616 | 3796 | 0.52 | 707 |
| HQ21 | X_55 | 100.3903 | 32.2128 | 3817 | 1.92 | 678 |
| HQ22 | X_54 | 100.2856 | 31.6417 | 3519 | 5.40 | 635 |

Table S2 The differences in the secondary metabolites between distinct sampling groups

| Site | Anisodine (mg/g) | Anisodamine (mg/g) | Atropine (mg/g) |
| --- | --- | --- | --- |
| HQ | 1.72±0.48a | 0.36±0.18a | 1.45±0.67a |
| HH | 0.82±0.47b | 0.77±0.38b | 3.67±1.66b |
| All | 1.19±0.65 | 0.61±0.37 | 2.76±1.74 |

Note: HQ, Hengduan-Qilian Mountains; HH, Hengduan- Himalaya Mountains. Values within the same column followed by different letters indicate significant differences (Wilcoxon rank-sum test, P < 0.05).

Table S3 The relationships between microbial communities and environmental factors revealed by Mantel test.

|  | r | P |
| --- | --- | --- |
| **Geographical factor:** |  |  |
| Geographic distance | **0.30** | **< 0.001** |
| Altitude | 0.11 | 0.03 |
| **Climatic factors:** |  |  |
| MAT | **0.12** | **0.04** |
| MAP | **0.16** | **0.004** |
| **Soil properties:** |  |  |
| TN | 0.05 | 0.25 |
| NO_3_ | -0.001 | 0.47 |
| NH_4_ | 0.007 | 0.43 |
| TP | 0.02 | 0.37 |
| AP | **0.21** | **0.01** |
| SOC | 0.04 | 0.27 |
| **Root fungal beta diversity:** |  |  |
| All | -0.06 | 0.80 |
| HH-enriched | **0.26** | **< 0.001** |
| HQ-enriched | **0.26** | **< 0.001** |
| **Root fungal alpha diversity:** |  |  |
| All | **0.14** | **0.03** |
| HH-enriched | **0.23** | **< 0.001** |
| HQ-enriched | **0.24** | **< 0.001** |
| **Root bacterial beta diversity:** |  |  |
| All | -0.10 | 0.86 |
| HH-enriched | **0.15** | **0.04** |
| HQ-enriched | 0.06 | 0.19 |
| **Root bacterial alpha diversity:** |  |  |
| All | -0.00002 | 0.47 |
| HH-enriched | -0.01 | 0.56 |
| HQ-enriched | 0.08 | 0.14 |

Table S4 Topological parameters of networks

|  | Bacteria | Fungi |
| --- | --- | --- |
| Number of nodes | 473 | 178 |
| Number of edges | 10485 | 956 |
| Average degree | 44.334 | 10.742 |
| Diameter | 5 | 9 |
| Average path length | 2.24 | 3.399 |
| Average Clustering Coefficient | 0.406 | 0.497 |
| Modularity | 0.358 | 0.582 |


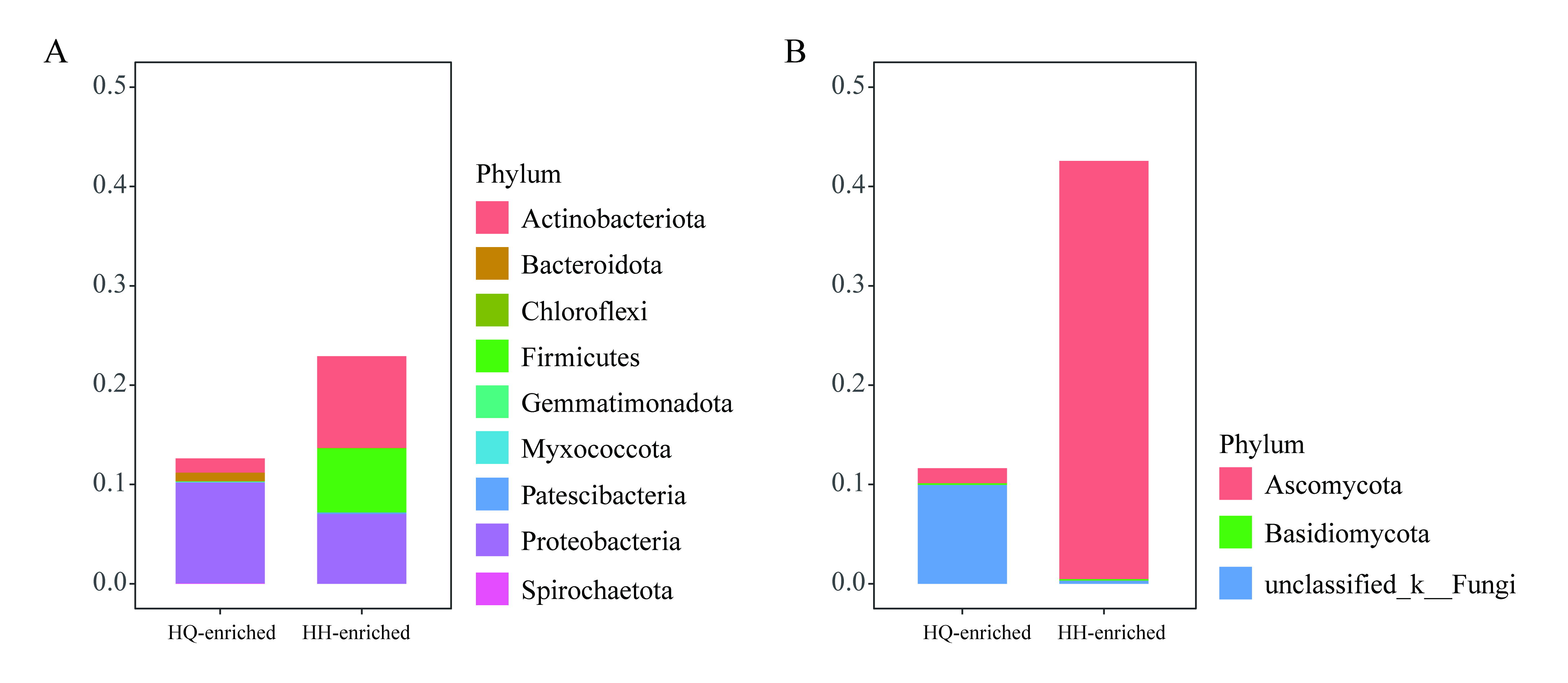


Fig. S1 Relative abundance of bacteria and fungi enriched in HQ and HH groups at the phyla level.


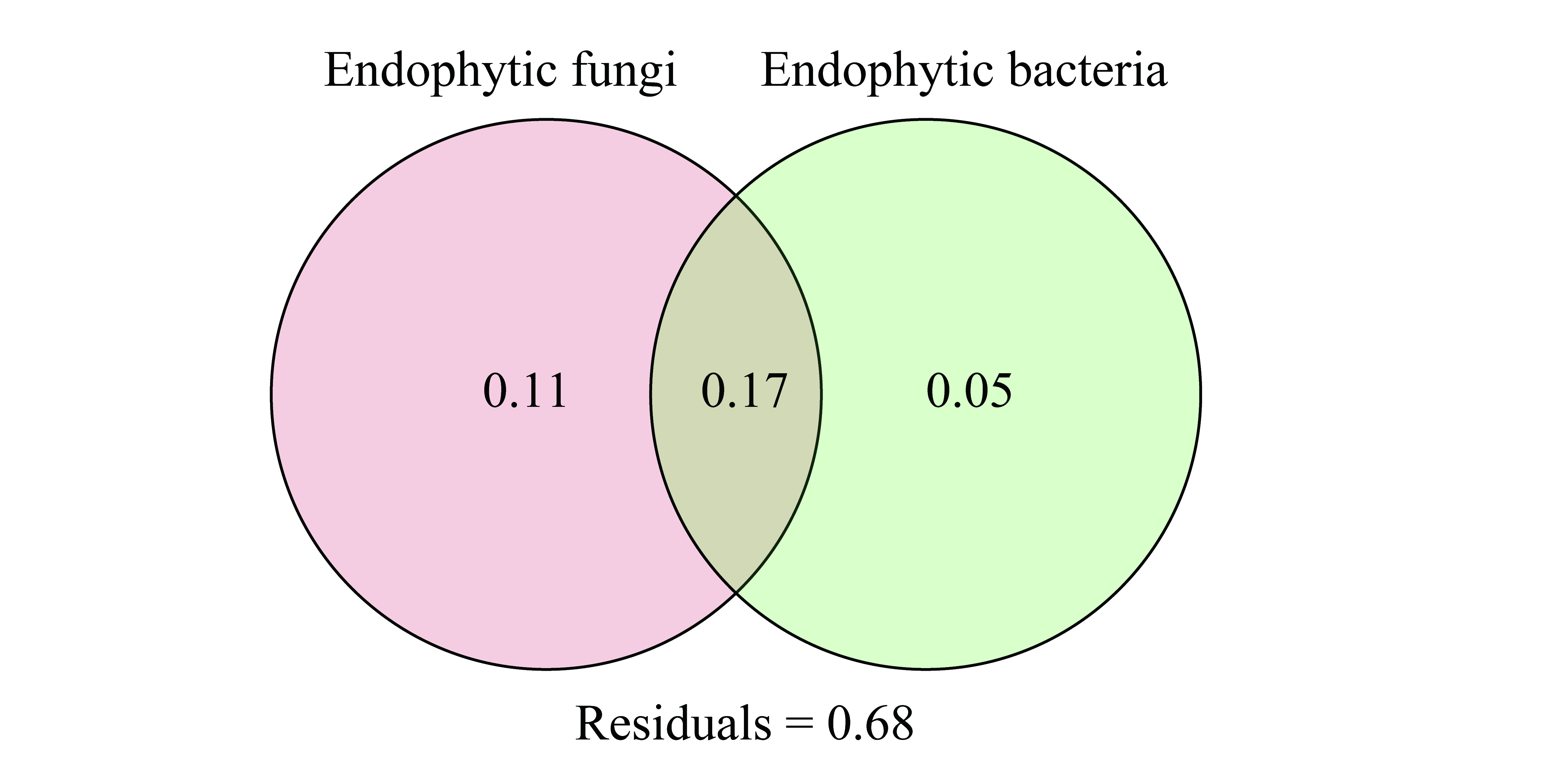


Fig. S2 Variation partitioning analysis (VPA) differentiate the contributions of endophytic fungi and endophytic bacteria on secondary metabolites variations. Numbers indicate the proportion of explained variation and residuals indicate unexplained variations.


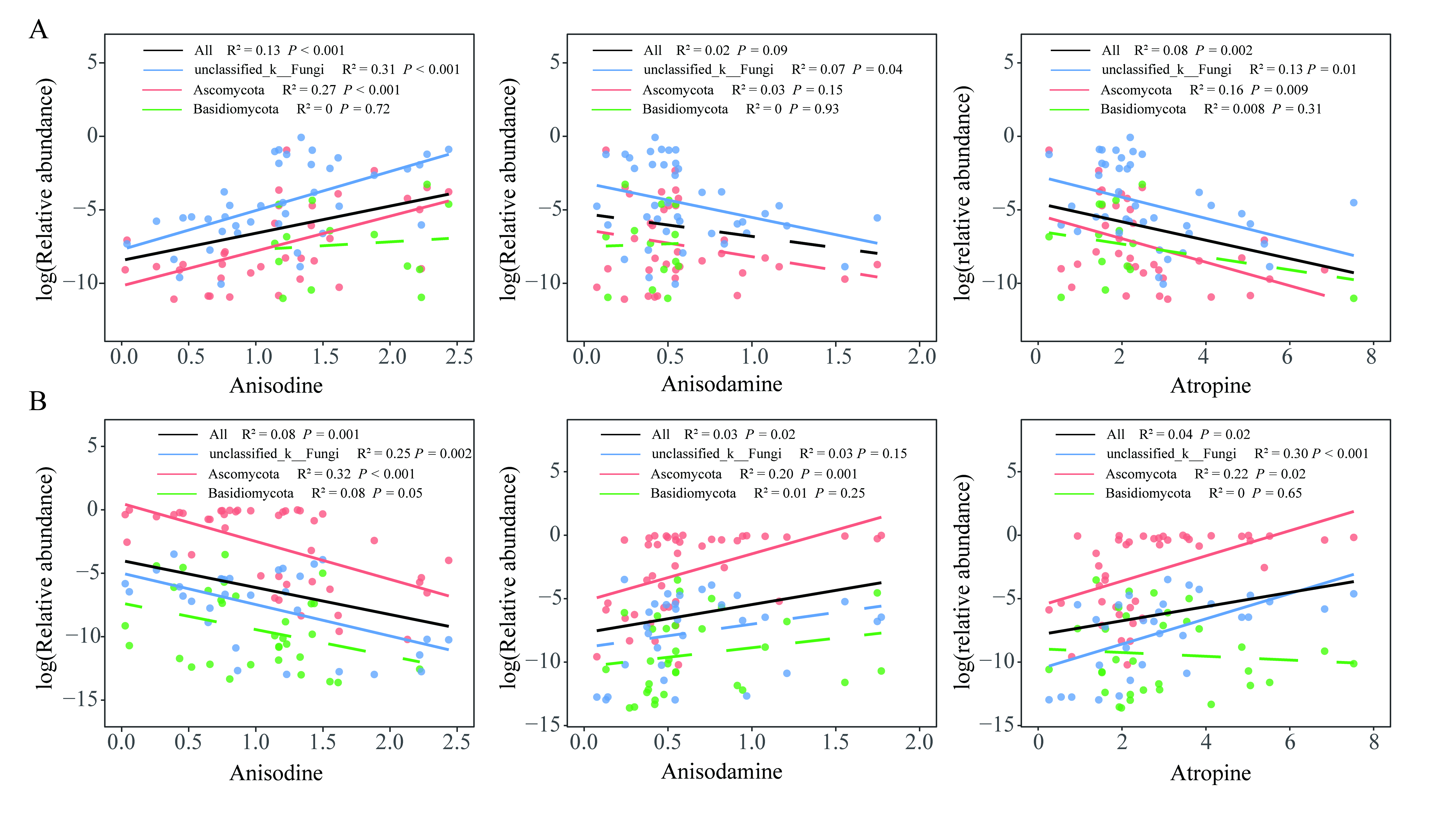


Fig. S3 (A) Regression analysis of relative abundance of enriched endophytic fungi in HQ group and three tropane alkaloids content. (B) Regression analysis of relative abundance of enriched endophytic fungi in HH group and three tropane alkaloids content.


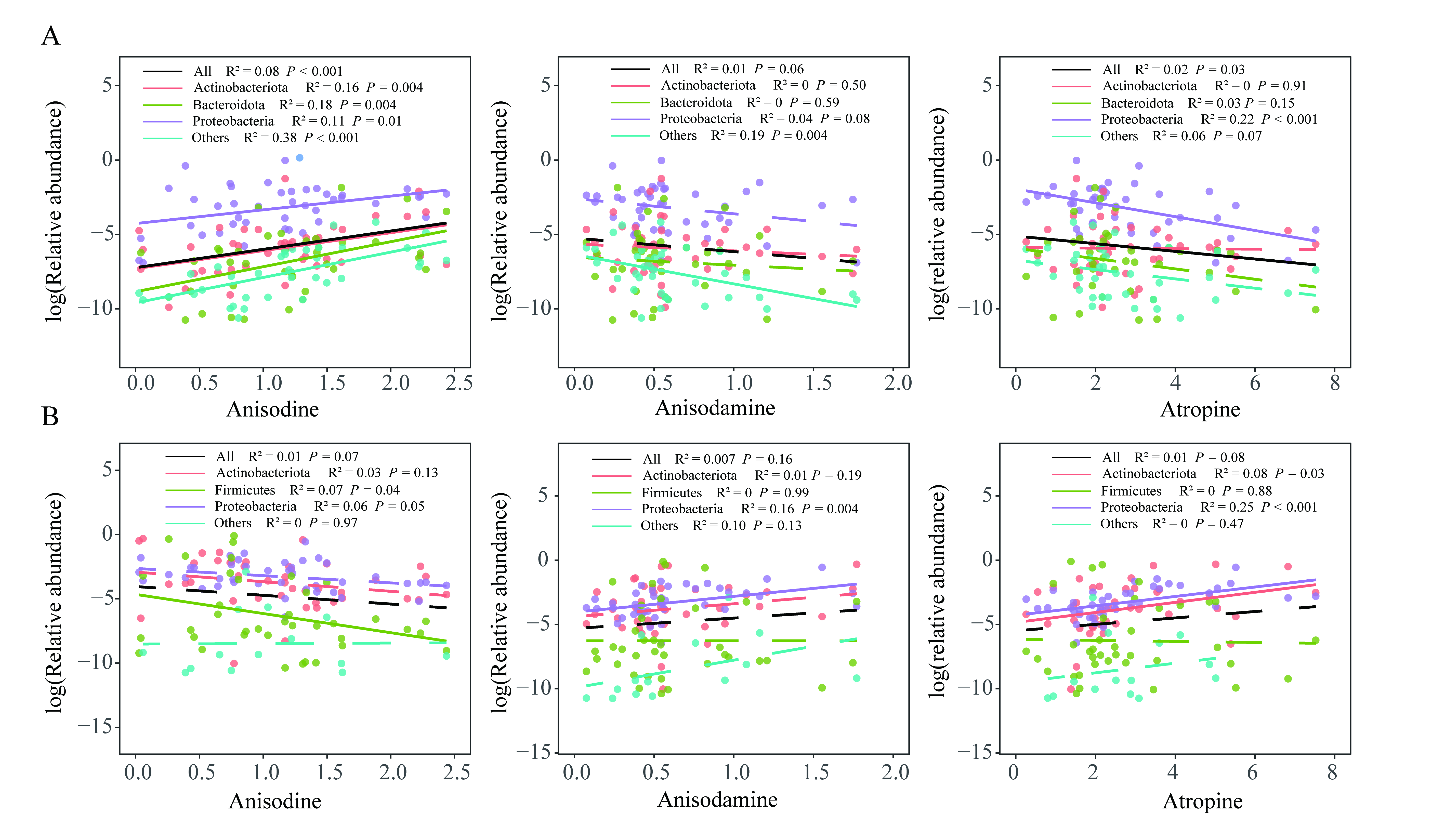


Fig. S4 (A) Regression analysis of relative abundance of enriched endophytic bacteria in HQ group and three tropane alkaloids content. (B) Regression analysis of relative abundance of enriched endophytic bacteria in HH group and three tropane alkaloids content.
